# Higher body mass index is associated with an increased risk of multiplicity in surveillance colonoscopy within 5 years

**DOI:** 10.1038/s41598-017-14163-9

**Published:** 2017-10-27

**Authors:** Chung Hyun Tae, Chang Mo Moon, Sung-Ae Jung, Chang Soo Eun, Jae Jun Park, Geom Seog Seo, Jae Myung Cha, Sung Chul Park, Jaeyoung Chun, Hyun Jung Lee, Yunho Jung, Jin Oh Kim, Young-Eun Joo, Sun-Jin Boo, Dong Il Park

**Affiliations:** 1 Department of Health Promotion Medicine, College of Medicine, Ewha Womans University, Seoul, Republic of Korea; 2 Department of Internal Medicine, College of Medicine, Ewha Womans University, Seoul, Republic of Korea; 3 Department of Internal Medicine, Hanyang University Guri Hospital, Guri, Republic of Korea; 4 Department of Internal Medicine, Gangnam Severance Hospital, Yonsei University College of Medicine, Seoul, Republic of Korea; 5 Department of Internal Medicine, Digestive Disease Research Institute, Wonkwang University College of Medicine, Iksan, Republic of Korea; 6 Department of Internal Medicine, Kyung Hee University Hospital at Gang Dong, Kyung Hee University School of Medicine, Seoul, Republic of Korea; 7 Department of Internal Medicine, Kangwon National University School of Medicine, Chuncheon, Republic of Korea; 8 Department of Internal Medicine and Liver Research Institute, Seoul National University College of Medicine, Seoul, Republic of Korea; 9 Department of Internal Medicine, Yonsei University College of Medicine, Seoul, Republic of Korea; 10 Department of Internal Medicine, Soonchunhyang University College of Medicine, Cheonan Hospital, Cheonan, Republic of Korea; 11 Department of Internal Medicine, Soonchunhyang University College of Medicine, Seoul Hospital, Seoul, Republic of Korea; 12 Department of Internal Medicine, Chonnam National University Medical School, Gwangju, Republic of Korea; 13 Department of Internal Medicine, Jeju National University School of Medicine, Jeju, Republic of Korea; 14 Department of Internal Medicine, Kangbuk Samsung Hospital, Sungkyunkwan University School of Medicine, Seoul, Republic of Korea

## Abstract

We aimed to evaluate whether obesity was associated with a certain clinicopathologic characteristics of metachronous CRA. This retrospective longitudinal cohort study included 2,904 subjects who had at least one resected CRA at index colonoscopy and who subsequently underwent one or more surveillance colonoscopies within 5 years. Of the 2,904 subjects, 60.9% (n = 1,769) were normal, 35.8% (n = 1,040) were overweight, and 3.3% (n = 95) were obese. Patients with any metachronous CRA were 53.7% (n = 1,559). In multivariate analyses, higher BMI at index colonoscopy was significantly associated with any metachronous CRA (overweight, OR = 1.07; obese, OR = 1.82; *p* for trend = 0.049). Regarding the multiplicity, the ORs of ≥ 3, ≥ 4 and ≥ 5 metachronous CRAs significantly increased as index BMI increased (*p* for trend < 0.001, = 0.007 and = 0.004, respectively). In negative binomial regression regarding the incidence for total number of metachronous CRA, the higher BMI the subject has at the time of index colonoscopy, the more metachronous CRAs the subject will have at the surveillance colonoscopy (*p* for trend = 0.016). Higher index BMI was significantly associated with the risk of multiple metachronous CRAs on surveillance colonoscopy within 5 years.

## Introduction

Epidemiologic studies have consistently reported an association between colorectal cancer (CRC) and obesity^1,2^. The incidence of CRC was significantly greater in obese patients of either sex^3^. Obesity is associated with a 30–70% increased risk of CRC and an increased risk of death from CRC^1,4,5^. Some studies have reported that weight loss after bariatric surgery or physical activity helped reduce the risk of CRC-related mortality^6,7^. In addition, several studies have suggested a close link between obesity and colorectal adenoma (CRA)^8–13^. Obesity increased the risk of CRA in both sexes and among ethnically diverse populations^14^. Furthermore, it has been reported that obesity increases the risk of metachronous CRA^2,15,16^. Physical activity could decrease the risk of CRA through negative feedback related to low-grade inflammation^17^. Although the role of obesity in CRA or CRC pathophysiology has not been fully elucidated, obesity contributes to progression from CRA to CRC. This progression could occur through adipokine secretion, chronic low-grade inflammation, insulin, and insulin-like growth factors related to obesity^5^.

However, there have not been any studies to evaluate which characteristics of metachronous colorectal lesions were influenced by index obesity in the longitudinal study. Therefore, we aimed to determine whether index obesity is associated with metachronous CRA in terms of prevalence, multiplicity, and advanced adenoma (AA) on surveillance colonoscopy within 5 years in the longitudinal cohort study.

## Results

### Baseline characteristics of subjects and index colonoscopy findings according to index body mass index

The clinical and index colonoscopy findings by body mass index (BMI) are summarized in Table 1. The mean age of the study population was 57.5 ± 9.0 years, and 60.9% (n = 1,769) were normal weight, 35.8% (n = 1,040) were overweight, and 3.3% (n = 95) were obese. Age, current smoking, and family history of CRC did not differ among BMI groups. However, sex, use of aspirin or nonsteroidal anti-inflammatory drugs (NSAIDs), and index colonoscopy findings significantly differed. BMI was not associated with the prevalence of low risk adenoma (LRA) or high risk adenoma (HRA) at the index colonoscopy (*p* = 0.164). However, in the HRA group, subjects with higher BMI were likely to have ≥3 non-AA (NAA) (*p* = 0.011) but not AA (*p* = 0.463). Subjects with higher BMI had more any CRAs (*p* = 0.001). When any CRA was classified into both NAA and AA categories, subjects with higher BMI were likely to have more NAAs (*p* = 0.003) but not more AAs (*p* = 0.107). The locations of any CRAs, NAAs, and AAs were not associated with BMI.

**Table 1:** Baseline characteristics of subjects and index colonoscopy findings according to index body mass index.

	Overall (n = 2,904)	Index BMI (kg/m^2^)			*p* for trend
		<25 (n = 1,769, 60.9%)	25–29 (n = 1,040, 35.8%)	≥30 (n = 95, 3.3%)	
Age (years), mean ± SD	57.5 ± 9.0	57.8 ± 9.1	57.2 ± 9.1	56.1 ± 8.6	0.059^†^
Sex, n (%)					<0.001^*^
Men	2,071 (71.3)	1,208 (68.3)	802 (77.1)	61 (64.2)	
Women	833 (28.7)	561 (31.7)	238 (22.9)	34 (35.8)	
Current smoking, n (%)	541 (18.6)	322 (18.2)	198 (19.0)	21 (22.1)	0.609^*^
Family history of CRC, n (%)	97 (3.4)	59 (3.4)	34 (3.4)	4 (4.3)	0.901^*^
Use of aspirin or NSAIDs, n (%)	377 (13.0)	201 (11.4)	152 (14.6)	24 (25.3)	<0.001^*^
Index colonoscopic findings					
Prevalence, n (%)					
LRA	1,693 (58.3)	1,055 (59.6)	587 (56.4)	51 (53.7)	0.164^*^
HRA	1,211 (41.7)	714 (40.4)	453 (43.6)	44 (46.3)	
≥3 NAA	655 (22.6)	368 (20.8)	259 (24.9)	28 (29.5)	0.011^*^
AA	773 (26.6)	462 (26.1)	289 (27.8)	22 (23.2)	0.463^*^
Number, mean ± SD					
Any CRA	2.3 ± 2.1	2.2 ± 2.0	2.5 ± 2.3	2.4 ± 1.8	0.001^**^
NAA	1.9 ± 1.9	1.9 ± 1.8	2.1 ± 2.0	2.1 ± 1.6	0.003^**^
AA	0.3 ± 0.7	0.3 ± 0.6	0.4 ± 0.7	0.3 ± 0.6	0.107^**^
Location, n (%)					
Any CRAs					0.158^*^
Right colon	909 (31.4)	550 (31.2)	333 (32.2)	26 (27.4)	
Left colon	1,193 (41.2)	751 (42.6)	407 (39.3)	35 (36.8)	
Both	792 (27.4)	463 (26.2)	295 (28.5)	34 (35.8)	
NAA					0.290^*^
Right colon	922 (32.7)	556 (32.5)	338 (33.4)	28 (30.1)	
Left colon	1,157 (41.1)	724 (42.3)	399 (39.4)	34 (36.6)	
Both	739 (26.2)	432 (252)	276 (27.2)	31 (33.3)	
AA					0.386^*^
Right colon	239 (32.0)	136 (30.3)	93 (33.9)	10 (41.7)	
Left colon	441 (59.0)	277 (61.7)	152 (55.5)	12 (50.0)	
Both	67 (9.0)	36 (8.0)	29 (10.6)	2 (8.3)	

BMI, body mass index; SD, standard deviation; CRC, colorectal cancer; NSAIDs, nonsteroidal anti-inflammatory drugs; LRA, low risk adenoma; HRA, high risk adenoma; NAA, non-advanced adenoma; AA, advanced adenoma; CRA, colorectal adenoma. ^*^Values by Cochran-Armitage trend for categorical variables. ^**^Values by linear regression. ^†^Values by One-way ANOVA analyses of variances for continuous variables among groups.

### Surveillance colonoscopy findings according to index BMI

The average follow-up period was 3.0 years, and patients underwent an average of 1.3 surveillance colonoscopies. The follow-up period (*p* = 0.102) and frequency of surveillance colonoscopy (*p* = 0.238) did not differ significantly among BMI groups. The characteristics of metachronous CRAs detected in surveillance colonoscopies were compared among BMI groups (Table 2). As index BMI increased, the proportion of subjects with any CRA increased significantly (*p* = 0.014). An increase across index BMI groups was positively associated with higher incidence of ≥3 NAA (*p* < 0.001), ≥3 any CRAs (*p* < 0.001), ≥4 any CRAs (*p* < 0.001) and ≥5 any CRAs (*p* < 0.001) in surveillance colonoscopy, but not with the incidence of AA (*p* = 0.936). In terms of CRA number, the total numbers of any CRAs and NAAs significantly increased as index BMI increased (*p* = 0.001 for any CRAs; *p* = 0.001 for NAA). However, the number of AAs (*p* = 0.977) and the incidence of CRC (*p* = 0.231) did not change significantly as index BMI increased. The locations of any CRAs (*p* = 0.487), NAAs (*p* = 0.750), and AAs (*p* = 0.637) did not differ among BMI categories. To summarize the univariate analyses, higher BMI was likely to have a significant association with multiple metachronous CRAs on surveillance colonoscopy.

**Table 2:** Surveillance colonoscopy findings according to index body mass index.

	Total (n = 2,904)	Index BMI (kg/m^2^)			*p* for trend
		<25 (n = 1,769, 60.9%)	25–29 (n = 1,040, 35.8%)	≥30 (n = 95, 3.3%)	
Incidence, n (%)					
Any CRAs	1,559 (53.7)	920 (52.0)	577 (55.5)	62 (65.3)	0.014^*^
LRA	987 (36.4)	606 (36.7)	347 (35.7)	34 (38.6)	0.789^*^
HRA	381 (14.0)	197 (11.9)	163 (16.8)	21 (23.9)	<0.001^*^
Any AA	191 (6.6)	117 (6.6)	67 (6.4)	7 (7.4)	0.936^*^
≥3 NAA	468 (16.1)	242 (13.7)	201 (19.3)	25 (26.3)	<0.001^*^
≥3 any CRAs	514 (17.7)	273 (15.4)	215 (20.7)	26 (27.4)	<0.001^*^
≥4 any CRAs	316 (10.9)	165 (9.3)	135 (13.0)	16 (16.8)	<0.001^*^
≥5 any CRAs	207 (7.1)	103 (5.8)	92 (8.8)	12 (12.6)	<0.001^*^
Number, mean ± SD					
Any CRAs	1.4 ± 2.1	1.3 ± 2.1	1.5 ± 2.2	1.8 ± 2.1	0.001^**^
NAA	1.3 ± 2.0	1.2 ± 2.0	1.4 ± 2.1	1.7 ± 2.0	0.001^**^
AA	0.1 ± 0.3	0.1 ± 0.3	0.1 ± 0.3	0.1 ± 0.3	0.977^**^
Location, n (%)					
Any CRAs					0.487^*^
Right colon	715 (41.0)	442 (61.8)	250 (35.0)	23 (3.2)	
Left colon	463 (26.6)	271 (58.5)	172 (37.1)	20 (4.3)	
Both	565 (32.4)	325 (57.5)	215 (38.1)	25 (4.4)	
NAA					0.750^*^
Right colon	688 (41.6)	421 (61.2)	242 (35.2)	25 (3.6)	
Left colon	503 (30.4)	293 (58.3)	188 (37.4)	22 (4.4)	
Both	461 (27.9)	266 (57.7)	175 (38.0)	20 (4.3)	
AA					0.637^*^
Right colon	325 (75.2)	211 (64.9)	101 (31.1)	13 (4.0)	
Left colon	95 (22.0)	59 (62.1)	33 (34.7)	3 (3.2)	
Both	12 (2.8)	6 (50.0)	6 (50.0)	0	
CRC incidence, n (%)	37 (1.3)	27 (1.5)	10 (1.0)	0	0.231^*^
Follow-up period (years), mean ± SD	3.0 ± 1.1	2.9 ± 1.1	3.0 ± 1.1	2.9 ± 1.0	0.102^†^
Frequency of surveillance colonoscopy, mean ± SD	1.3 ± 0.5	1.2 ± 0.5	1.3 ± 0.5	1.3 ± 0.6	0.238^†^

BMI, body mass index; CRA, colorectal adenoma; LRA, low risk adenoma; HRA, high risk adenoma; AA, advanced adenoma; NAA, non-advanced adenoma; SD, standard deviation; CRC, colorectal cancer. ^*^Values by Cochran-Armitage trend for categorical variables. ^**^Values by linear regression. ^†^Values by One-way ANOVA analyses of variances for continuous variables among groups.

### Association of index BMI with the characteristics of metachronous CRA

Next, to evaluate the association between index BMI and the risk of metachronous CRA, univariate analyses (Supplementary Tables 1 and 2) and subsequent logistic multivariate analyses (Table 3 and Supplementary Table 2) were performed. Obese subjects with ≥30 kg/m^2^ had a significant association with the presence of any metachronous CRA (adjusted OR, 1.82; 95% CI, 1.15−2.89) compared to those with <25 kg/m^2^. There was a statistically significant trend in increased adjusted OR of any CRA as increasing BMI (*p* for trend = 0.049) (Table 3). When stratified by sex, both obese men and women had higher risk of metachronous CRA (adjusted OR, 1.77; 95% CI, 1.01−3.09 for men; adjusted OR, 2.30; 95% CI, 1.08−4.90 for women) compared to those with normal BMI, but did not reach statistical significance (*p* for trend = 0.061 for men and 0.075 for women) (Table 4).

**Table 3:** Association between clinicopathologic characteristics and metachronous colorectal adenomas.

	Logistic multivariate analyses								Negative binomial regression	
	Any CRAs (n = 1,559) (vs. Absent CRA, n = 1, 345)		≥ 3 any CRAs (n = 514) (vs. < 3, n = 2,390)		≥ 4 any CRAs (n = 316) (vs. < 4, n = 2,588)		≥ 5 any CRAs (n = 207) (vs. < 5, n = 2,697)		Metachronous CRAs (n = 2,904)	
	aOR (95% CI)	*p* value	aOR (95% CI)	*p* value	aOR (95% CI)	*p* value	aOR (95% CI)	*p* value	Exp (B) (95% CI)	*p* value
Age	1.02 (1.01–1.03)	<0.001	1.03 (1.02–1.05)	<0.001	1.30 (1.02–1.05)	<0.001	1.04 (1.02–1.06)	<0.001	1.02 (1.01–1.02)	<0.001
Men	1.87 (1.56–2.24)	<0.001	2.62 (1.96–3.51)	<0.001	2.89 (1.97–4.24)	<0.001	3.20 (1.92–5.32)	<0.001	1.68 (1.48–1.90)	<0.001
Index BMI (kg/m^2^)										
<25	1.00 (reference)		1.00 (reference)		1.00 (reference)		1.00 (reference)		1.00 (reference)	
25–29	1.07 (0.91–1.26)	0.432	1.34 (1.08–1.67)	0.009	1.35 (1.03–1.77)	0.031	1.46 (1.05–2.04)	0.024	1.08 (0.97–1.20)	0.158
≥30	1.82 (1.15–2.89)	0.011	2.23 (1.31–3.80)	0.003	1.93 (1.01–3.69)	0.048	2.39 (1.13–5.06)	0.023	1.40 (1.07–1.84)	0.016
*p* for trend		0.049		<0.001		0.007		0.004		0.016
Current smoking	1.23 (0.99–1.52)	0.058	1.25 (0.96–1.62)	0.079	1.08 (0.78–1.49)	0.638	1.30 (0.89–1.91)	0.172	1.14 (1.00–1.30)	0.048
Family history of CRC	1.27 (0.82–2.00)	0.284	1.13 (0.62–2.08)	0.681	1.16 (0.55–2.45)	0.705	1.09 (0.42–2.81)	0.860	1.04 (0.79–1.39)	0.762
Use of aspirin or NSAIDs	1.08 (0.85–1.37)	0.517	1.01 (0.75–1.37)	0.956	1.38 (0.97–1.96)	0.071	1.36 (0.89–2.08)	0.158	1.05 (0.09–1.22)	0.544
Index colonoscopy										
LRA	1.00 (reference)	<0.001	1.00 (reference)	<0.001	1.00 (reference)	<0.001	1.00 (reference)	<0.001	1.00 (reference)	
HRA	1.80 (1.53–2.12)		2.29 (1.84–2.84)		2.71 (2.06–3.56)		2.99 (2.12–4.23)		1.76 (1.58–1.95)	<0.001
Follow-up period	1.13 (1.05–1.22)	0.002	1.16 (1.05–1.29)	0.005	1.16 (1.02–1.33)	0.026	1.25 (1.06–1.48)	0.008	1.12 (1.06–1.18)	<0.001
Frequency of surveillance	2.58 (2.11–3.16)	<0.001	3.10 (2.55–3.77)	<0.001	3.54 (2.84–4.40)	<0.001	3.64 (2.85–4.64)	<0.001	1.87 (1.71–2.06)	<0.001

CRA,colorectal adenoma; aOR, adjusted odds ratio; 95% CI, 95% confidence interval; BMI, body mass index; CRC, colorectal cancer; NSAIDs, nonsteroidal anti-inflammatory drugs; LRA, low risk adenoma; HRA, high risk adenoma. Hosmer-Lemeshow Goodness-of-Fit test of each model showed *p* = 0.552 for any CRAs, *p* = 0.887 for ≥3 any CRAs, *p* = 0.470 for ≥4 any CRAs, and *p* = 0.427 for ≥5 any CRAs.

**Table 4:** Association between index body mass index and metachronous colorectal adenomas stratified by sex.

Index BMI (kg/m^2^)	Logistic multivariate analyses							
	Any CRAs (n = 1,559) (vs. Absence, n = 1,345)		≥3 any CRAs (n = 514) (vs. <3, n = 2,390)		≥4 any CRAs (n = 316) (vs. <4, n = 2,588)		≥5 any CRAs (n = 207) (vs. <5, n = 2,697)	
	n (% of total)	aOR (95% CI)	n (% of total)	aOR (95% CI)	n (% of total)	aOR (95% CI)	n (% of total)	aOR (95% CI)
Overall								
<25	920 (52.0)	1.00 (reference)	273 (15.4)	1.00 (reference)	165 (9.3)	1.00 (reference)	103 (5.8)	1.00 (reference)
25–29	577 (55.5)	1.07 (0.91–1.26)	215 (20.7)	1.34 (1.08–1.67)	135 (13.0)	1.35 (1.03–1.77)	92 (8.8)	1.46 (1.05–2.04)
≥30	62 (65.3)	1.82 (1.15–2.89)	26 (27.4)	2.23 (1.31–3.80)	16 (16.8)	1.93 (1.01–3.69)	12 (12.6)	2.39 (1.13–5.06)
*p* for trend		0.049		<0.001		0.007		0.004
Men								
<25	701 (58.0)	1.00 (reference)	229 (19.0)	1.00 (reference)	143 (11.8)	1.00 (reference)	93 (7.7)	1.00 (reference)
25–29	473 (59.0)	1.01 (0.90–1.30)	194 (24.2)	1.43 (1.12–1.81)	124 (15.5)	1.41 (1.05–1.88)	85 (10.6)	1.47 (1.04–2.09)
≥30	42 (68.9)	1.77 (1.01–3.09)	21 (34.4)	2.38 (1.28–4.40)	12 (19.7)	2.08 (0.76–3.49)	9 (14.8)	1.89 (0.79–4.51)
*p* for trend		0.061		<0.001		0.015		0.016
Women								
<25	219 (39.0)	1.00 (reference)	44 (7.8)	1.00 (reference)	22 (3.9)	1.00 (reference)	10 (1.8)	—^*^
25–29	104 (43.7)	1.12 (0.81–1.54)	21 (8.8)	1.00 (0.56–1.78)	11 (4.6)	0.97 (0.44–2.11)	7 (2.9)	—^*^
≥30	20 (58.8)	2.30 (1.08–4.90)	5 (14.7)	1.86 (0.62–5.64)	4 (11.8)	2.72 (0.79–9.45)	3 (8.8)	—^*^
*p* for trend		0.075		0.504		0.334		—^*^

CRA, colorectal adenoma; AA, advanced adenoma; BMI, body mass index; n, number; aOR, adjusted odd ratio; 95% CI, 95% confidence interval. Adjusted for age, index BMI, current smoking, family history of colorectal cancer, use of aspirin or nonsteroidal anti-inflammatory drugs, LRA or HRA at index, follow-up period, and frequency of surveillance colonoscopy in men and women analyses. Additionally adjusted by sex in overall analyses. Hosmer-Lemeshow Goodness-of-Fit test of each model showed *p* = 0.702 for any CRAs in men, *p* = 0.100 for any CRAs in women, *p* = 0.656 for ≥3 any CRAs in men, *p* = 0.143 for ≥3 any CRAs in women, *p* = 0.844 for ≥4 any CRAs in men, *p* = 0.916 for ≥4 any CRAs in women, *p* = 0.930 for ≥5 any CRAs in men, and *p* = 0.662 for ≥5 any CRAs in women. ^*^For ≥5 any CRAs in women, we couldn’t calculate adjusted ORs and 95% CI because of <30 cases.

For multiplicity of metachronous CRAs, the adjusted ORs for ≥3 metachronous CRAs stepwise increased across increasing BMI categories (adjusted OR, 1.34; 95% CI, 1.08−1.67 for subjects with 25−30 kg/m^2^; adjusted OR, 2.23, 95% CI, 1.31−3.80 for subjects with >30 kg/m^2^; *p* trend <0.001) (Table 3). Likewise, the adjusted OR for ≥4 CRAs (adjusted OR, 1.35; 95% CI, 1.03−1.77 for subjects with 25−30 kg/m^2^; adjusted OR, 1.93; 95% CI, 1.01−3.69 for subjects with >30 kg/m^2^; *p* trend = 0.007), ≥5 CRAs (adjusted OR, 1.46; 95% CI, 1.05−2.04 for subjects with 25−30 kg/m^2^; adjusted OR, 2.39; 95% CI, 1.13−5.60 for subjects with >30 kg/m^2^; *p* trend = 0.004) increased stepwise across increasing BMI categories (Table 3). In negative binomial regression regarding the incidence for total number of metachronous CRA, the higher BMI the subject has at the time of index colonoscopy, the more metachronous CRAs the subject will have at the surveillance colonoscopy (p for trend = 0.016) **(**Table 3 **)**. When stratified by sex (Table 4 **)**, adjusted ORs increased significantly in men (*p* for trend <0.001, < 0.015 and 0.016 for ≥3, ≥4, and ≥5 metachronous CRAs, respectively). However, among women, BMI was not significantly associated with ≥3 and ≥4 metachronous CRAs. Too few women had ≥5 any metachronous CRAs to calculate the adjusted OR (n < 30). In addition, the incidence of metachronous AAs was not significantly associated with BMI in analyses overall (Supplementary Table 2).

## Discussion

This large longitudinal cohort study demonstrated a dose-dependent association between index BMI and the multiplicity of metachronous CRAs on surveillance within 5 years after the index colonoscopy. This association between index BMI and the risk of multiple CRAs persisted in analyses of men, but not in women.

Many previous studies have reported an association between obesity and CRAs in cross-sectional design and meta-analysis^2,8,9,11,12,16^. In addition, some longitudinal studies have reported that obesity was related to metachronous CRAs^10,13,15,18^. However, all of them have focused that obesity is associated with prevalence of CRAs or metachronous CRAs, but didn’t concern about a certain specific feature having metachronous CRAs^2,10,15,16,19^. To date, studies on specific characteristics of metachronous CRAs related to obesity are scarce and inconsistent. One study investigated the relationship between BMI and CRA growth by following-up *in situ* over 3 years, which suggested that adenoma growth was associated with BMI^13^. However, the result of these positive associations was only from analyses of 13 cases. It didn’t seem not be enough to test statistical assumptions. Meanwhile, another study found no difference among obesity and non-obesity groups in size of metachronous CRAs in spite of large pooling study including 1,213 subjects^2^. With regard to tumor location, a pooled analysis of seven prospective studies found an association between obesity and metachronous CRAs in proximal colon^18^. However, another prospective study found no differences among obesity and non-obesity group according to CRA location^2^.

In the present study, we focused on the effect of increased index BMI on multiple metachronous CRAs. To our knowledge, three studies have evaluated an association between BMI and multiple metachronous CRAs. Among them, two studies have demonstrated no difference between BMI and multiple metachronous CRAs. One study with 163 Japanese autopsy subjects demonstrated that the mean number of polyps had no significant trend with increasing BMI quartile^20^. In another longitudinal study of 119 subjects, monotonic trends between various indices representing obesity and metachronous CRAs were inconsistent^21^. However, the former study was not only relatively small, but also an observational study that did not adjust for other possible risk factors for multiple metachronous CRAs. The latter study had a limited sample size for conducting stratified number of metachronous CRAs. To our knowledge, there is only one clinical study of 8,213 subjects consisting of nearly 90% Caucasian reporting an increased risk for 1> any metachronous CRAs in obese men^18^. This report have demonstrated that risk for 1> any metachronous CRAs were increased with OR 1.4 in obese men with BMI ≥ 30 kg/m^2^ compared to those of subjects with normal BMI, which finding is consistent with our results^18^. Even though 1,904 subjects was all Asian and proportion of obesity with ≥30 kg/m^2^ was lower than those of the former study, we have clearly shown that increasing index BMI in a dose-response manner is a strong predictor for developing multiple metachronous CRAs. In subgroup analyses, the OR of ≥3 metachronous CRAs significantly increased according to index BMI category (BMI <25, 25−29, ≥30 kg/m^2^). In addition, we demonstrated that obesity was not associated with AA at index data, corresponding to both cross-sectional and longitudinal data. Therefore, we suggest the obesity significantly affects the formation of colorectal tumor, but not metachronous AA^22^. Molecular studies have shown that insulin, insulin-like growth factor (IGF-1) axis, adipokines, sex-steroid hormones, and chronic low-grade inflammation are the main pathways linking obesity and colorectal neoplasia, including CRAs and CRC. Some experimental studies support the involvement of obesity in initiating colorectal tumorigenesis. An adiponectin deficiency was associated with the development of early colorectal neoplasm rather than advanced CRC^23,24^. Adipose tissues express higher quantities of pro-inflammatory molecules such as TNF-α, IL-6, CRP^25^. Elevated pro-inflammatory markers, including CRP and prostaglandin E2, are considered to be biomarkers of multiple CRAs or AA risk^26^.

Our results demonstrated a difference in the effect of obesity on metachronous CRAs between genders. BMI was associated with multiple metachronous CRAs in men but not in women. In a study with similar results about this issue, little heterogeneous association among gender with a stronger relation for men alike our results^18^. There has been similarly observed gender difference of BMI impact in the association for CRC risk or CRA prevalence^1,2,13,16,27^. A strong association with adenoma risk among men than women has been reported^10,16,19^. A cohort study stratified by age reported an association between BMI and cancer risk in premenopausal but not postmenopausal women^27^. We performed further analyses stratified by age (stratified at 50 years as the mean menopause age in Korean women), but did not find a significant association between BMI and multiple metachronous CRAs in women. These results are probably related to both the small number of women and the lower incidence of overall CRAs than in men. This weak association between obesity and CRC in women has been explained in part by the protective action of estrogen, as suggested by epidemiologic studies^28^. At least three hormonal axes, insulin-like growth factors, insulin, and estrogen, might be relevant in obesity and CRC risk in women^28,29^. Obesity increases the risk of CRC through insulin and the insulin-like growth factors axis^29^. In postmenopausal women, a major source of estrogen is adipose tissue. Insulin and insulin-like growth factors and estrogen seem to have opposite influences in postmenopausal women^27,28^. The difference in associations between BMI and CRAs between men and women could be influenced by the different distribution of central body fat^30^.

Our study is limited by several factors. First, we could not assess actual fluctuations in BMI over the follow-up period. Our study design was based on index BMI. Weight gain or loss over 5 year period might be an effect. Nevertheless, previous studies showed short-term weight change over 4 year period and weight gain and loss did not affect metachronous CRAs^9–11,15^. Second, this was not a population-based study, but a unique cohort study that included participants who underwent surveillance colonoscopies after index colonoscopy at academic institutions across the country. Therefore, our study was subject to selection bias, and generalization is limited. Third, our data consistently demonstrated that obesity was not related to the risk for metachronous AA. This finding contrasts with some previous reports, which found increasing BMI to be associated with the risk of metachronous AA^10,16,19^. Our non-significant trend might be originated from relatively short follow-up duration and lower prevalence of AA than those in West^31^.

Nevertheless, the main strength of our study is the large longitudinal cohort design. To date, several cross- sectional studies have shown a relationship between obesity and CRAs. These results could not demonstrate causality between obesity and CRAs due to limitation of study design. We suggested the new feature of multiplicity of metachronous CRAs related to obesity. We applied multivariate analyses, the trend for linearity to assess the dose-response relationship, and negative binomial regression between BMI and multiple metachronous CRAs.

In conclusion, this study identified that increased index BMI is associated with increased risk of multiple metachronous CRAs. This phenomenon significant remained in subgroup analyses for men but not women. Further studies are necessary to confirm our results and identify the underlying mechanisms in multiple CRAs in obese subjects. Higher index BMI may be a considerable clinical factor in determining of post-poylpectomy surveillance strategy.

## Methods

### Subjects

We conducted a retrospective longitudinal cohort study using a multicenter polyp surveillance registry of the Korean Association for the Study of Intestinal Disease. Data were pooled from 3,687 subjects who underwent an index colonoscopy, from 2006 to 2008, who subsequently underwent at least one or more surveillance colonoscopies. Indications for index colonoscopy were screening of CRC, anemia, rectal bleeding, constipation, diarrhea, weight loss, abdominal pain, and positive fecal occult blood test. Subjects were included if they met any of the following criteria: 1) those who underwent a qualified index colonoscopy; 2) whose polyps were removed at the index colonoscopy; 3) who had at least one CRA that was histologically confirmed; and 4) those between 30 and 85 years of age. In this study, qualified index colonoscopy was defined when cecal intubation was successful, all colorectal polyps were completely removed, and the bowel preparation was adequate. In addition, all colonoscopies were performed by experienced endoscopists who were professors or fellows at university hospitals and had performed more than 500 supervised colonoscopies.

To exclude the possibility of missing polyps at the qualified index colonoscopy, we considered an adenoma to be missed at the index colonoscopy if any adenoma was detected on a colonoscopy within 6 months after the index colonoscopy. To evaluate the effect of BMI on metachronous CRAs, subjects who underwent the first surveillance colonoscopy at least 2 years after the index colonoscopy were selected to address the potential reverse causation of both BMI and metachronous CRA. To minimize the impact of BMI change over time, subjects who underwent at least one surveillance colonoscopy within 2 to 5 years from the index colonoscopy were included.

**Figure 1:**
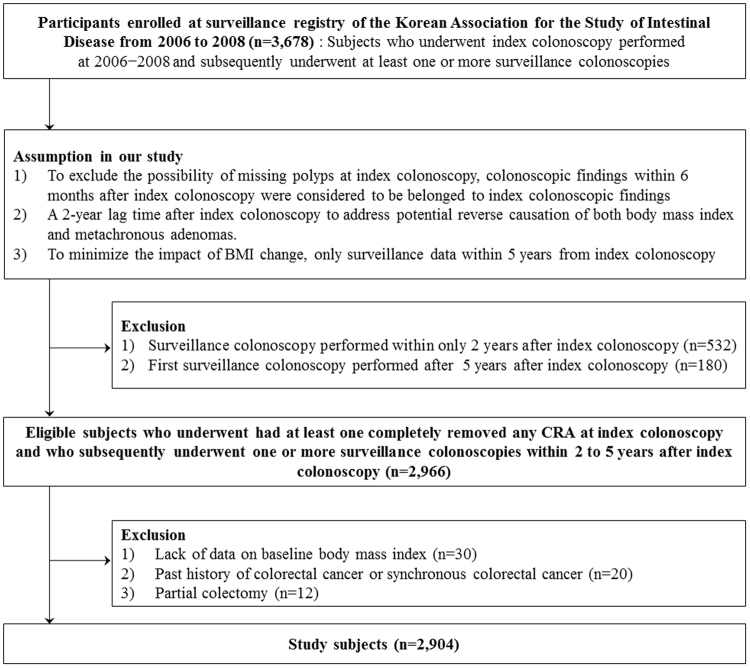
Flow diagram of study subjects.

The exclusion criteria were as follows: 1) lack of BMI data at the time of index colonoscopy (n = 30); 2) surveillance colonoscopy performed within only 2 years after index colonoscopy (n = 532); 3) first surveillance colonoscopy performed more than 5 years after index colonoscopy (n = 180); 4) past history of CRC or synchronous CRC (n = 20); and 5) previous partial colectomy (n = 12). Finally, a total of 2,904 subjects were included in this study **(**Fig. 1). This study was approved by the Institutional Review Board of the Ewha Womans University Mokdong Hospital (EUMC 2015-04-041). Due to the retrospective nature of the study, informed consent was waived. All the experimental procedures were conducted in accordance with institutional ethical guidelines. This study has been carried out in accordance with the Declaration of Helsinki.

### Data collection and definitions

Medical history and clinical data were obtained from patient’s medical records. These data included age at index colonoscopy, sex, body weight and height at the time of index colonoscopy. BMI was calculated as weight in kilograms divided by the square of the height in meters. Subjects were categorized into 3 groups according to baseline BMI: < 25 kg/m^2^ (normal weight), 25–29 kg/m^2^ (overweight), and ≥ 30 kg/m^2^ (obese). Current smoking, family history of CRC, and use of aspirin or NSAIDs during the previous 12 months were also collected.

CRA characteristics at index and surveillance colonoscopies were collected from endoscopy and pathology reports. CRAs were stratified into LRA and HRA groups. LRA was defined as one or two non-AAs. HRA was further classified as any AA or 3 or more non-AAs. AA was defined as any polyp with one or more of the following features: ≥ 10 mm, villous histology, or high-grade dysplasia. Subjects simultaneously diagnosed with AA and non-AA were classified into AA group. If all CRAs were located in ascending colon, hepatic flexure, or transverse colon, the location of the lesion was defined as ‘right colon’. If all CRAs were located in splenic flexure, descending colon, sigmoid colon, or rectum, the location of the lesion was defined as ‘left colon’. If adenomas were present in both the right and left colon, the location was ‘both sided’.

### Statistical analyses

Data entry and statistical analyses were performed with the Statistical Package for the Social Science (SPSS) version 20.0 (SPSS Inc., Chicago, Illinois, US). Index and surveillance colonoscopy findings were summarized as mean and standard deviation for continuous variables and frequency and percentage for categorical variables. The analysis was stratified by index BMI. Linear regression for continuous variables and Cochran-Armitage analyses for categorical variables were used to assess the linear trend. For the age, follow-up period, frequency of surveillance colonoscopy of continuous variables, we used the One-way ANOVA analyses for variance among patients.

Logistic multivariate analyses were conducted to ascertain the impact of index BMI on metachronous CRAs. Adjusted odds ratio (OR) and 95% confidence interval (CI) for BMI categories were calculated separately for subjects with vs without any CRAs, < 2 vs ≥ any CRAs, < 3 vs ≥ 3 any CRAs, < 4 vs ≥ 4 any CRAs, < 5 vs ≥ 5 CRAs, and with or without any AA. The Hosmer-Lemeshow Goodness-of-Fit test was used to assess the calibration of the each model in logistic multivariate analyses. However, good calibration ability are not sufficient for a model about ≥ 2 any CRAs to be clinically useful. Therefore, the data relevant to ≥ 2 any CRAs were excluded. In addition, we compared the incidence for total number of metachronous CRA as continuous, not binary variables. We used negative binomial regression for number of metachronous CRAs because the assumption of equality of the mean and variance in the Poisson model did not hold true. In all statistical models, we adjusted for age, sex, index BMI status, current smoking, family history of CRC, use of aspirin or NSAID, index colonoscopic findings, follow-up period, and frequency of surveillance.

## Electronic supplementary material

Supplementary Information
