# Supplementary material for: Higher body mass index is associated with an increased risk of multiplicity in surveillance colonoscopy within 5 years
Source: Sci Rep. 2017 Oct 27;7:14239. doi: 10.1038/s41598-017-14163-9 (PMC5660255; doi:10.1038/s41598-017-14163-9)
Supplement: Supplementary file 1 — Supplementary Information [file 41598_2017_14163_MOESM1_ESM.doc]

**Supplementary information**

**Higher body mass index is associated with an increased risk of multiplicity in surveillance colonoscopy within 5 years**

Chung Hyun Tae^1^, Chang Mo Moon^2*^, Sung-Ae Jung^2^, Chang Soo Eun^3^, Jae Jun Park^4^, Geom Seog Seo^5^, Jae Myung Cha^6^, Sung Chul Park^7^, Jaeyoung Chun^8^, Hyun Jung Lee^9^, Yunho Jung^10^, Jin Oh Kim^11^, Young-Eun Joo^12^, Sun-Jin Boo^13^, Dong Il Park^14^

^1^Department of Health Promotion Medicine, College of Medicine, Ewha Womans University, Seoul, Republic of Korea

^2^Department of Internal Medicine, College of Medicine, Ewha Womans University, Seoul, Republic of Korea

^3^Department of Internal Medicine, Hanyang University Guri Hospital, Guri, Republic of Korea

^4^Department of Internal Medicine, Gangnam Severance Hospital, Yonsei University College of Medicine, Seoul, Republic of Korea

^5^Department of Internal Medicine, Digestive Disease Research Institute, Wonkwang University College of Medicine, Iksan, Republic of Korea

^6^Department of Internal Medicine, Kyung Hee University Hospital at Gang Dong, Kyung Hee University School of Medicine, Seoul, Republic of Korea

^7^Department of Internal Medicine, Kangwon National University School of Medicine, Chuncheon, Republic of Korea

^8^Department of Internal Medicine and Liver Research Institute, Seoul National University College of Medicine, Seoul, Republic of Korea

^9^Department of Internal Medicine, Yonsei University College of Medicine, Seoul, Republic of Korea

^10^Department of Internal Medicine, Soonchunhyang University College of Medicine, Cheonan Hospital, Cheonan, Republic of Korea

^11^Department of Internal Medicine, Soonchunhyang University College of Medicine, Seoul Hospital, Seoul, Republic of Korea

^12^Department of Internal Medicine, Chonnam National University Medical School, Gwangju, Republic of Korea

^13^Department of Internal Medicine, Jeju National University School of Medicine, Jeju, Republic of Korea

^14^Department of Internal Medicine, Kangbuk Samsung Hospital, Sungkyunkwan University School of Medicine, Seoul, Republic of Korea

**Supplementary Table 1. Association between the clinicopathologic characteristics and metachronous colorectal adenomas**: univariate analyses

|  | Metachronous CRAs | | | | | | | | | | | |
| --- | --- | --- | --- | --- | --- | --- | --- | --- | --- | --- | --- | --- |
|  | Absence | Presence | *p* value | < 3 | ≥ 3 | *p* value | <4 | ≥4 | *p* value | <5 | ≥ 5 | *p* value |
| Age (years), mean ± SD | 56.6 ± 9.4 | 58.4 ± 8.6 | <0.001 | 57.1 ± 9.0 | 59.4 ± 9.0 | <0.001 | 57.3 ± 9.0 | 60.0 ± 9.2 | <0.001 | 57.3 ± 9.0 | 60.4 ± 8.8 | <0.001 |
| Men, n (%) | 855 (63.6) | 1,216 (78.0) | <0.001 | 1,627 (68.1) | 444 (86.4) | <0.001 | 1,792 (69.2) | 279 (88.3) | <0.001 | 1,884 (69.9) | 187 (90.3) | <0.001 |
| Index BMI (kg/m^2^), n (%) |  |  | 0.007 ^*^ |  |  | <0.001* |  |  | 0.002 |  |  | <0.001 * |
| < 25 | 849 (63.1) | 920 (52.0) |  | 1,496 (62.6) | 273 (53.1) |  | 1604 (62.0) | 165 (52.2) |  | 1,666 (61.8) | 103 (49.8) |  |
| 25-29 | 463 (34.4) | 577 (37.0) |  | 825 (34.5) | 215 (41.8) |  | 905 (35.0) | 135 (42.7) |  | 948 (35.2) | 92 (44.4) |  |
| ≥ 30 | 33 (2.5) | 62 (4.0) |  | 69 (2.9) | 26 (5.1) |  | 79 (3.1) | 16 (5.1) |  | 83 (3.1) | 12 (5.8) |  |
| Current smoker, n (%) | 208 (15.7) | 333 (21.5) | <0.001 | 413 (17.5) | 128 (23.7) | <0.001 | 464 (18.1) | 77 (24.4) | 0.007 | 485 (18.2) | 56 (27.2) | 0.001 |
| Family history of CRC, n (%) | 44 (3.4) | 53 (3.5) | 0.864 | 81 (3.5) | 16 (3.2) | 0.741 | 87 (3.4) | 10 (3.2) | 0.862 | 91 (3.4) | 6 (3.0) | 0.746 |
| Use of aspirin or NSAIDs, n (%) | 154 (11.4) | 223 (14.3) | 0.022 | 300 (12.6) | 77 (15.0) | 0.137 | 320 (12.4) | 57 (18.0) | 0.005 | 339 (12.6) | 38 (18.4) | 0.017 |
| Index colonoscopy, n (%) |  |  | <0.001 |  |  | <0.001 |  |  | <0.001 |  |  | <0.001 |
| LRA | 911 (67.7) | 782 (50.2) |  | 1,500 (62.8) | 193 (37.5) |  | 1,594 (61.6) | 99 (31.3) |  | 1,637 (60.7) | 56 (27.1) |  |
| HRA | 434 (32.3) | 777 (49.8) |  | 890 (37.2) | 321 (62.5) |  | 994 (38.4) | 217 (68.7) |  | 1,060 (39.3) | 151 (72.9) |  |
| Follow-up period (years), mean ± SD | 2.8 ± 1.1 | 3.1 ± 1.1 | <0.001 | 2.9 ± 1.1 | 3.2 ± 1.1 | <0.001 | 2.9 ± 1.1 | 3.3 ± 1.1 | <0.001 | 2.9 ± 1.1 | 3.4 ± 1.0 | <0.001 |
| Frequency of surveillance, mean ± SD | 1.1 ± 0.3 | 1.4 ± 0.6 | <0.001 | 1.2 ± 0.4 | 1.6 ± 0.8 | <0.001 | 1.2 ± 0.4 | 1.7 ± 0.9 | <0.001 | 1.2 ± 0.4 | 1.9 ± 0.9 | <0.001 |

CRA, colorectal adenoma; AA, advanced adenoma; SD, standard deviation; BMI, body mass index; CRC, colorectal cancer; NSAIDs, nonsteroidal anti-inflammatory drugs; LRA, low risk adenoma; HRA, high risk adenoma. ^*^ Values by Cochran-Armitage trend for categorical variables.

**Supplementary Table 2. Association between the clinicopathologic characteristics and metachronous**

|  | Any AAs (n = 191)  (vs. Absence, n = 2,713) | | | | |
| --- | --- | --- | --- | --- | --- |
|  | Univariate | | | Logistic multivariate | |
|  | Absence | Presence | *p* value | aOR (95% CI) | *p* value |
| Age (years), mean ± SD | 58.4 ± 9.1 | 59.5 ± 8.8 | 0.002 | 1.02 (1.01-1.04) | 0.027 |
| Men, n (%) | 1918 (70.7) | 153 (80.1) | 0.005 | 1.59 (1.09-2.34) | 0.017 |
| Index BMI (kg/m^2^), n (%) |  |  | 0.936 |  |  |
| < 25 | 1652 (60.9) | 117 (61.3) |  | 1.00 (reference) |  |
| 25-29 | 973 (35.9) | 67 (35.1) |  | 0.89 (0.64-1.23) | 0.481 |
| ≥ 30 | 88 (3.2) | 7 (3.7) |  | 1.04 (0.46-2.35) | 0.935 |
| *p* for trend |  |  |  |  | 0.617 |
| Current smoker, n (%) | 510 (19.0) | 31 (16.3) | 0.362 | 0.69 (0.45-1.05) | 0.086 |
| Family history of CRC, n (%) | 88 (3.3) | 9 (4.8) | 0.284 | 1.69 (0.81-3.46) | 0.168 |
| Use of aspirin or NSAIDs, n (%) | 349 (12.9) | 28 (14.7) | 0.475 | 0.99 (0.64-1.55) | 0.968 |
| Index colonoscopy, n (%) |  |  | <0.001 |  | <0.001 |
| LRA | 1623 (59.8) | 70 (36.6) |  | 1.00 (reference) |  |
| HRA | 1090 (40.2) | 121 (63.4) |  | 2.10 (1.53-2.88) |  |
| Follow-up period (years), mean ± SD | 2.9 ± 1.1 | 3.1 ± 1.1 | <0.001 | 0.97 (0.83-1.13) | 0.650 |
| Frequency of surveillance, mean ± SD | 1.2 ± 0.5 | 1.5 ± 0.8 | 0.126 | 2.02 (1.59-2.57) | <0.001 |

**advanced adenomas: univariate and logistic multivariate analyses**

AA, advanced adenoma; SD, standard deviation; BMI, body mass index; CRC, colorectal cancer; NSAIDs, nonsteroidal anti-inflammatory drugs; LRA, low risk adenoma; HRA, high risk adenoma. Hosmer-Lemeshow Goodness-of-Fit test of model showed *p* = 0.228 for any AA.
